# Mismatch Repair Protein Msh6^Tt^ Is Necessary for Nuclear Division and Gametogenesis in *Tetrahymena thermophila*

**DOI:** 10.3390/ijms242417619

**Published:** 2023-12-18

**Authors:** Lin Wang, Sitong Yang, Yuhuan Xue, Tao Bo, Jing Xu, Wei Wang

**Affiliations:** 1 Key Laboratory of Chemical Biology and Molecular Engineering of Ministry of Education, Institute of Biotechnology, Shanxi University, Taiyuan 030006, China; 201913002004@email.sxu.edu.cn (L.W.); yangsitong77@163.com (S.Y.); 202223002021@email.sxu.edu.cn (Y.X.); botao@sxu.edu.cn (T.B.); 2 Shanxi Key Laboratory of Biotechnology, Taiyuan 030006, China; 3 School of Life Science, Shanxi University, Taiyuan 030006, China

**Keywords:** *Tetrahymena thermophila*, mismatch repair protein Msh6^Tt^, nuclear division, gametogenesis

## Abstract

DNA mismatch repair (MMR) improves replication accuracy by up to three orders of magnitude. The MutS protein in *E. coli* or its eukaryotic homolog, the MutSα (Msh2-Msh6) complex, recognizes base mismatches and initiates the mismatch repair mechanism. Msh6 is an essential protein for assembling the heterodimeric complex. However, the function of the Msh6 subunit remains elusive. *Tetrahymena* undergoes multiple DNA replication and nuclear division processes, including mitosis, amitosis, and meiosis. Here, we found that Msh6^Tt^ localized in the macronucleus (MAC) and the micronucleus (MIC) during the vegetative growth stage and starvation. During the conjugation stage, Msh6^Tt^ only localized in MICs and newly developing MACs. *MSH6^Tt^* knockout led to aberrant nuclear division during vegetative growth. The *MSH6^Tt^KO* mutants were resistant to treatment with the DNA alkylating agent methyl methanesulfonate (MMS) compared to wild type cells. *MSH6^Tt^* knockout affected micronuclear meiosis and gametogenesis during the conjugation stage. Furthermore, Msh6^Tt^ interacted with Msh2^Tt^ and MMR-independent factors. Downregulation of *MSH2^Tt^* expression affected the stability of Msh6^Tt^. In addition, *MSH6^Tt^* knockout led to the upregulated expression of several *MSH6^Tt^* homologs at different developmental stages. Msh6^Tt^ is involved in macronuclear amitosis, micronuclear mitosis, micronuclear meiosis, and gametogenesis in *Tetrahymena*.

## 1. Introduction

The integrity of DNA and stability of chromatin are essential for the accurate transmission of genetic information. Exogenous or endogenous stress causes DNA damage, threatening the stability of chromatin [1]. Endogenous genomic mutations are inevitably generated during physiological DNA processing, resulting in mismatches and insertion or deletion errors [2]. Some chemically modified nucleic acid precursors, such as 8-oxo-dGTP and dUTP, are misincorporated into DNA sequences by DNA polymerases during replication, causing replication-associated DNA damage [3]. This seriously violates Watson–Crick base pairing rules, causing DNA synthesis error rates to vary by more than a million-fold [4]. Specific DNA damage causes cancer, aging, and other diseases [5]. Organisms have developed various repair mechanisms to cope with DNA damage [1]. The combined action of DNA polymerase proofreading and DNA mismatch repair (MMR) are essential to ensure the accuracy of replication during each cell division [2]. The MMR system is responsible for repairing the mispairs that escape the proofreading activity of the polymerase after replication [4]. The evolutionarily conserved MMR system improves replication accuracy by up to three orders of magnitude through cut and resynthesis mechanisms during DNA replication, ensuring the integrity of genetic information [6]. The initiator of MMR is the MutS complex, which recognizes the mismatched base and then recruits the MutL complex to the mismatch site. The assembly of the MutL-MutS complex is sufficient to activate downstream repair activities [7]. MutS occurs as homodimers in *Escherichia coli* [8] and heterodimeric complexes in eukaryotic organisms [9]. MutS homodimers are functionally and structurally asymmetric [10,11,12]. Both subunits of *E. coli* MutS have the Phe-X-Glu motif that binds directly to DNA, and only one subunit interacts directly with mismatched bases. In contrast, the associated structural domains of the other subunit do not interact specifically with the DNA backbone [11]. In the human MutSα (Msh2-Msh6) and MutSβ (Msh2-Msh3) heterodimeric complexes, Msh3 or Msh6 mainly binds to mismatched bases [9]. Human cells generally express more *hMSH6* than *hMSH3*, resulting in an hMutSα:hMutSβ ratio of approximately 10:1 [13]. Bacterial MutS adopts an asymmetric conformation for ATP binding [12]. In the Msh2-Msh6 complex, the Msh6 subunit has a higher affinity for ATP (ten times higher than that of Msh2), and Msh2 has a higher affinity for ADP [14]. MutSα and MutSβ are involved in mismatch recognition, and their functions partially overlap [15]. MutSα is primarily responsible for repairing single base mismatches and single insertion/deletion mismatches [16]. MutSβ is mainly involved in repairing insertion/deletion mismatches and can repair up to 16 additional nucleotides in a single strand [16].

In addition to repairing replication errors, MMR proteins are involved in other DNA transactions, including the repair of double-stranded DNA breaks and DNA damage monitoring in response to apoptotic signaling [4]. In mouse and human cells, abundant programmed double-strand breaks occur during meiosis [17]. Double-strand break repair begins with programmed DNA double-strand breaks catalyzed by the Spo11 protein [18]. Homologous recombination uses homologous parental chromosome sequences to repair breaks, and if homologous recombination exchanges heterologous chromosome sequences, mismatches occur, and MMR is involved in completing the exchange of genetic information [19]. While meiotic recombination can tolerate a certain degree of heterologous sequences, mitotic recombination is quite sensitive to mismatches, and this sensitivity is dependent on the MMR mechanism [20]. MMR inhibits partial homologous recombination when heterologous DNA contains an excess of mismatched bases [19]. In *E. coli*, the mismatch repair-related MutS, MutL, and MutH, and the deconjugating enzyme UvrD inhibit partial homologous recombination [21]. Knockdown of Msh6 in yeast cells promotes the formation of the mitotic recombination product [22]. hMsh2-hMsh6 binds to Holliday junctions in human cells [23]. The MMR system functions as a detector that senses DNA damage and activates the apoptotic process. The absence of MMR-associated proteins leads to the expression of drug resistance in cells [24]. In mouse embryonic fibroblasts (MEFs), the knockdown of *MSH2* increases cellular tolerance to 6-thioguanine [25]. The interaction between yeast cellular MMR proteins and DNA polymerase δ suggests that MMR proteins operate at replication forks [26]. Histone assembly and mismatch repair mechanisms regulate each other [27]. The MMR mechanism can prevent chromosome assembly when mismatches are present [28]. In human cells, H3K36me3 recruits hMutSα to chromatin to ensure timely repair of DNA replication mismatches [29]. Overall, MutS, particularly Msh6, is a critical protein in the MMR-dependent DNA damage response and communication with other DNA repair pathways [30]. However, because of the complexity and transient nature of the function of MMR proteins [31], the detailed mechanism for establishing mismatch repair remains to be investigated.

*Tetrahymena thermophila* performs asexual proliferation and sexual reproduction [32]. During the vegetative proliferation stage, the micronucleus (MIC) divides mitotically, and the macronucleus (MAC) divides amitotically. During the conjugation stage, the MIC undergoes two meiotic divisions to form four identical haploid pronuclei, one of which is selected and undergoes mitosis to produce gametic nuclei. Haploid gametic nuclei are then reciprocally exchanged between mating partners and fertilized to form a diploid zygotic nucleus [32]. The zygotic nucleus then undergoes two postzygotic mitoses. The two anterior anlagen develop into new MACs, while the remaining two posterior products become MICs. The new MACs undergo large-scale genome rearrangement and amplification processes. The parental MAC is degraded by autophagy-induced programmed death [32]. Within the MAC and MIC, the chromatin states are distinct. The genetic material of *Tetrahymena* undergoes multiple divisions, including mitosis, meiosis, and amitosis [32].

Approximately 15,000 predicted protein-coding genes in *Tetrahymena* have intense matches to genes in other organisms [33]. Msh6 structural domains are conserved from bacteria to humans [34]. Here, the functions of Msh6^Tt^, a subunit of MutSα, were explored in *Tetrahymena*. Msh6^Tt^ localized in the MAC and MIC during the vegetative growth and starvation stage. During the conjugation stage, Msh6^Tt^ localized in the MICs and newly developing MACs. *MSH6^Tt^* knockout led to the accumulation of aberrant cells. The *MSH6^Tt^KO* mutants were resistant to methyl methanesulfonate (MMS) treatment. Furthermore, the stability of Msh6^Tt^ was dependent on Msh2^Tt^. Msh6^Tt^ interacted with Msh2^Tt^ and MMR-independent factors. *MSH6^Tt^* have various functional complementary genes at different developmental stages. Overall, Msh6^Tt^ was involved in macronuclear amitosis, micronuclear mitosis, and micronuclear meiosis. Msh6^Tt^ is necessary for gametogenesis and sexual reproduction.

## 2. Results

### 2.1. Characterization of MSH6^Tt^ in T. thermophila

**Figure 1:**
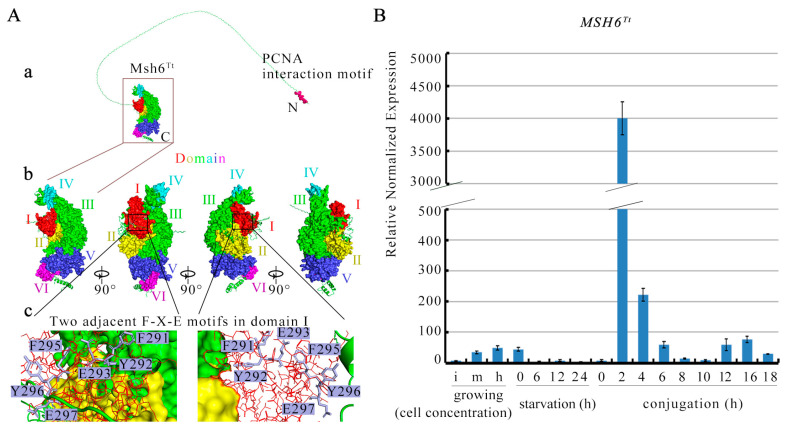
Characterization of *MSH6^Tt^* in *T. thermophila*. (**A**) Msh6^Tt^ is colored from domain I to domain VI with red, yellow, green, cyan, purple-blue, and hot pink. (**a**), The complete modeling of Msh6^Tt^ entire protein using Phyre 2 with intensive modeling mode. (**b**), Four views of Msh6^Tt^ related to 90° rotations as indicated. (**c**), Two adjacent F-X-E motifs in domain I. Structural domain I is shown as red lines, and the F-X-E motifs are shown as blue–white sticks. The amino acid positions of the two F-X-E motifs are 291–293 and 295–297. (**B**) The relative expression profiles of *MSH6^Tt^* at different development stages. The expression of *MSH6^Tt^* was analyzed by qRT-PCR. The *y*-axis indicates the relative expression of *MSH6^Tt^*. The data were normalized to expression of *MSH6^Tt^* at 6 h of starvation. For growing cells, i, m, and h correspond to ~1 × 10^5^ cells/mL, ~3.5 × 10^5^ cells/mL, and ~1 × 10^6^ cells/mL, respectively. For starvation, ~2 × 10^5^ cells/mL were collected at 0, 6, 12, and 24 h, referred to as starvation (h) 0, 6, 12, and 24. For conjugation, equal volumes of B2086 and CU428 cells were mixed, and samples were collected at 0, 2, 4, 6, 8, 10, 12, 16, and 18 h after mixing, referred to as conjugation (h) 0, 2, 4, 6, 8, 10, 12, 14, 16, and 18.

*MSH6^Tt^* (TTHERM_00194810) is the homologous gene of human *MSH6* and yeast *MSH6* in *Tetrahymena*. Msh6^Tt^ contains 1232 amino acids consisting of six conserved structural domains termed domains I to VI (6, 34) and an N-terminal disordered domain at amino acids 1-266 (Figure 1(Aa,Ab)). The conserved Pcna interaction motifs are contained in the N-terminal disordered region of Msh6^Tt^ (Figure 1(Aa)). The Phe-X-Glu motif (F-X-E motif) in domain I, which is conserved from *E. coli* to humans, is in direct contact with mispaired bases [6]. Two consecutive F-X-E motifs are specifically present in Msh6^Tt^ domain I, separated by only one amino acid (Figure 1(Ac)). During the vegetative growth, the expression level of *MSH6^Tt^* increased with increasing cell concentration. However, during starvation, the expression of *MSH6^Tt^* was low. The highest expression of *MSH6^Tt^* was observed at 2 h of conjugation. Subsequently, from 4 h to 10 h of conjugation, the expression of *MSH6^Tt^* gradually decreased. This dynamic regulation of *MSH6^Tt^* expression could be important in ensuring the integrity and accuracy of the genetic material exchanged in *T. thermophila* (Figure 1B, Table S2).

### 2.2. Msh6^Tt^-3HA Localizes in the MIC and MAC during Vegetative Growth and Starvation

**Figure 2:**
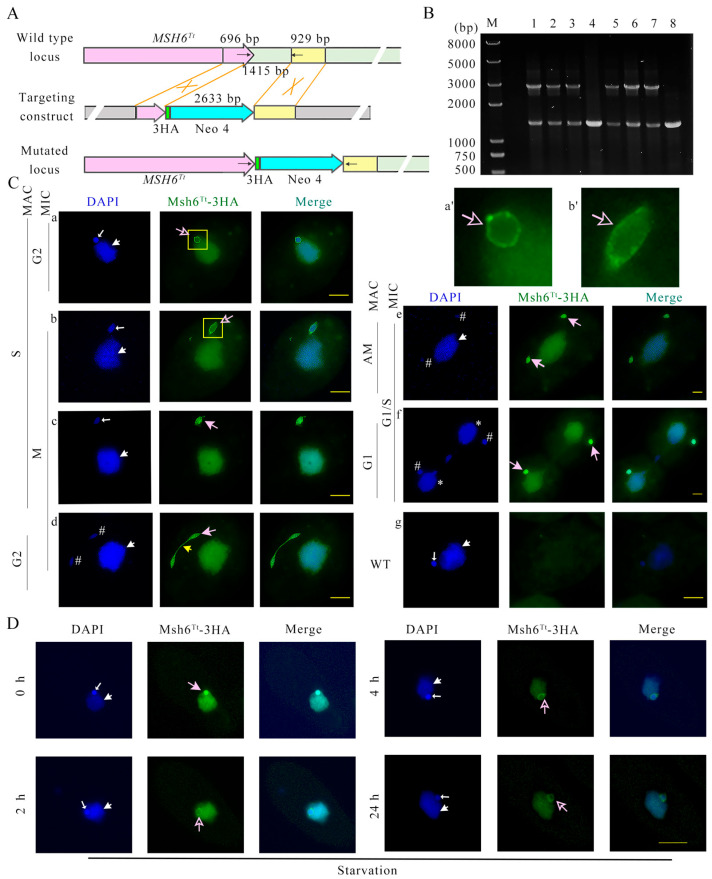
The localization of Msh*6^Tt^*-3HA in the amitotic MAC and mitotic MIC during vegetative proliferation and starvation stages. (**A**) Schematic representation of generating recombinant Msh*6^Tt^*-3HA mutants in *T. thermophila*. The pink arrow in the wild type (WT) locus indicates the *MSH6^Tt^*. The pink arrow and yellow box present in the targeting construct indicate the homologous arm. The 1415 bp green box is replaced by 3HA and Neo4 cassette. The black arrows indicate the position of the PCR primers when identifying the mutant cell line (the length of the arrow is independent of the primer length). (**B**) The identification of Msh*6^Tt^*-3HA-B2086 and Msh*6^Tt^*-3HA-CU428 mutants. M indicates marker; 1–3 indicate Msh*6^Tt^*-3HA-CU428 mutants; 4 and 8 indicate WT cells; 5–7 indicate Msh*6^Tt^*-3HA-B2086 mutants. The WT gene and mutation sites were amplified by PCR, which should be 2633 bp for the mutation site and 1415 bp for the WT gene. The black arrows in the figure (**A**) mark the positions of the identification primers in this PCR. (**C**) (**a**–**f**), Immunofluorescence localization of Msh*6^Tt^*-3HA in the amitotic MAC and mitotic MIC during vegetative proliferation. (**g**), WT negative control; (**a’**,**b’**) are five times the corresponding parts of a and b, respectively. The large white arrows indicate the MACs; the small white arrows indicate the MICs; the yellow arrow indicates that Msh*6^Tt^*-3HA forms a dumbbell shape and is localized in the circular region and the middle part. # indicates the new MICs after mitosis; * indicates the new MACs after amitosis. Hollow pink arrows indicate the localization of Msh6^Tt^-3HA in the perinuclear region of the MIC. The solid pink arrow indicates the localization of Msh6^Tt^-3HA in the MIC. (**D**) Immunofluorescence localization of Msh*6^Tt^*-3HA during starvation. The indirect immunofluorescence localization signal of Msh*6^Tt^*-3HA is green (HA label). DAPI staining of the nuclei is blue. The large white arrows indicate the MACs; the small white arrows indicate the MICs. The scale bar is 10 μm.

To explore localization of Msh6^Tt^, Msh6^Tt^-3HA mutants were constructed (Figure 2A,B and Figure S1). Msh6^Tt^-3HA localized in the MAC and MIC during the vegetative growth and starvation stage (Figure 2C,D). During vegetative proliferation, the MIC is most attached to the MAC. In the G1, S, and G2 phases of MIC, the position of the MIC on the MAC correlates with cell polarity, but before mitosis, the MIC is fixed at the “equator” of the MAC [35]. The fluorescence signals of Msh6^Tt^-3HA were stronger in the perinuclear area of the MIC during the G2 phase (Figure 2(Ca,Ca’)) and pre-M phase (Figure 2(Cb,Cb’)) of MIC. After MIC left the MAC, perinuclear localization disappeared, and Msh6^Tt^-3HA was localized into the MIC (Figure 2(Cc–Cf)). In addition, Msh6^Tt^-3HA formed a spindle-like structure during mitosis of MIC (Figure 2(Cb,Cc)), and in late mitosis of MIC, Msh6^Tt^-3HA formed a dumbbell shape and localized in both the circular region at each end and the middle part (Figure 2(Cd)). Msh6^Tt^-3HA localized in the MIC during early starvation, and the Msh6^Tt^-3HA signal was strengthened in the perinuclear area of MIC during 2 h to 24 h of starvation (Figure 2D).

### 2.3. Msh6^Tt^-3HA Localizes in the MIC and New MAC during Conjugation

**Figure 3:**
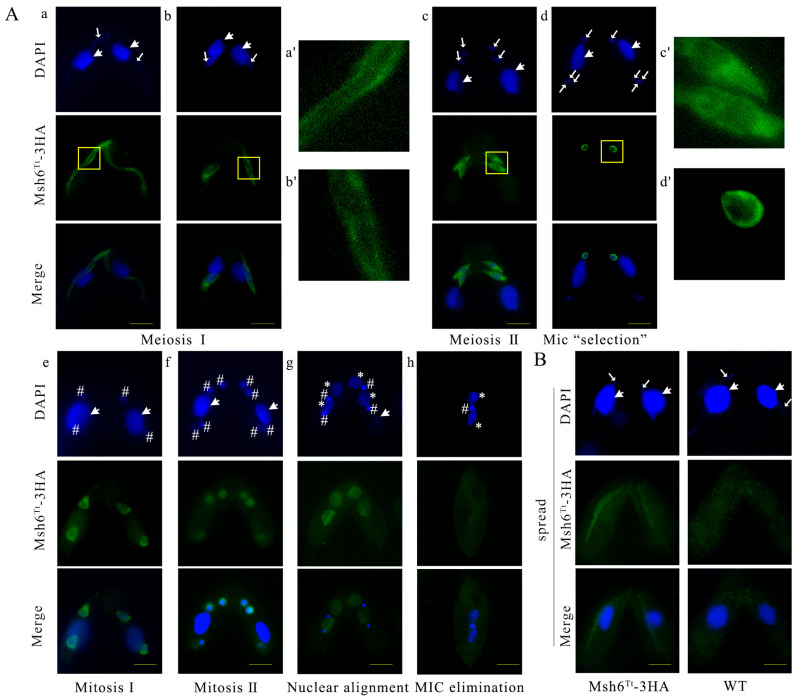
The localization of Msh*6^Tt^*-3HA in the MIC and new developing MAC during conjugation stage. (**A**) (**a**–**h**), Immunofluorescence localization of Msh*6^Tt^*-3HA during conjugation. (**a’**–**d’**) is five times the corresponding part (marked with a yellow box) of (**a**–**d**). (**B**) Msh*6^Tt^*-3HA localized on chromatin in spread cells. The chromatin-binding proteins will remain in the nucleus after using an enhanced detergent to spread cells. The detergent treatment disrupts non-specific interactions and removes loosely bound proteins from the chromatin. The large white arrows indicate the MACs; the small white arrows indicate the MICs. # indicates the new MICs; * indicates the new MACs. The indirect immunofluorescence localization signal of Msh*6^Tt^*-3HA is green (HA label). The DAPI staining of the nuclei is blue. The scale bar is 10 μm.

During the conjugation stage, the MIC undergoes two meiotic divisions to form four identical haploid pronuclei, one of which is selected and undergoes mitosis to produce gametic nuclei. At the end of MIC selection, three MICs are located at the posterior of the cell, and one MIC remains at the anterior [35]. Msh6^Tt^-3HA localized in the meiotic MIC (Figure 3(Aa–Ac)) and formed spindle-like structures (Figure 3(Ab,Ac)). After nuclear selection, Msh6^Tt^-3HA localized only to the selected pronuclei and not to the MICs that will be degraded (Figure 3(Ad)). The zygotic nucleus undergoes two mitotic divisions and forms two new MICs and two new MACs. Msh6^Tt^-3HA localized in the new postzygotic nuclei (Figure 3(Ae,Af)). However, the signal of Msh6^Tt^-3HA was stronger on two of the four postzygotic nuclei (Figure 3(Af)). Then, the localization of Msh6^Tt^-3HA in the newly developing MACs increased and disappeared in the new MICs (Figure 3(Ag)). When the *Tetrahymena* cells developed into exconjugants, the parental MAC initiated nuclear apoptosis. Since Msh6^Tt^-3HA was expressed from the parental MAC genome, the Msh6-3HA signal disappeared in the late conjugation stage (Figure 3(Ah)). During micronuclear meiosis, the signal of Msh6^Tt^-3HA was more robust in the perinuclear region than in the nucleus (Figure 3(Aa–Ac,Aa’–Ac’)). After nuclear selection, Msh6^Tt^-3HA localized around the selected MIC (Figure 3(Ad,Ad’)). After detergent treatment, the perinuclear localization of Msh6^Tt^-3HA disappeared but was maintained in the MICs (Figure 3B). The results showed that Msh6^Tt^ is involved in DNA replication and nuclear division.

### 2.4. MSH6^Tt^ Knockout Affects Nuclear Divisions during the Vegetative Growth Stage

**Figure 4:**
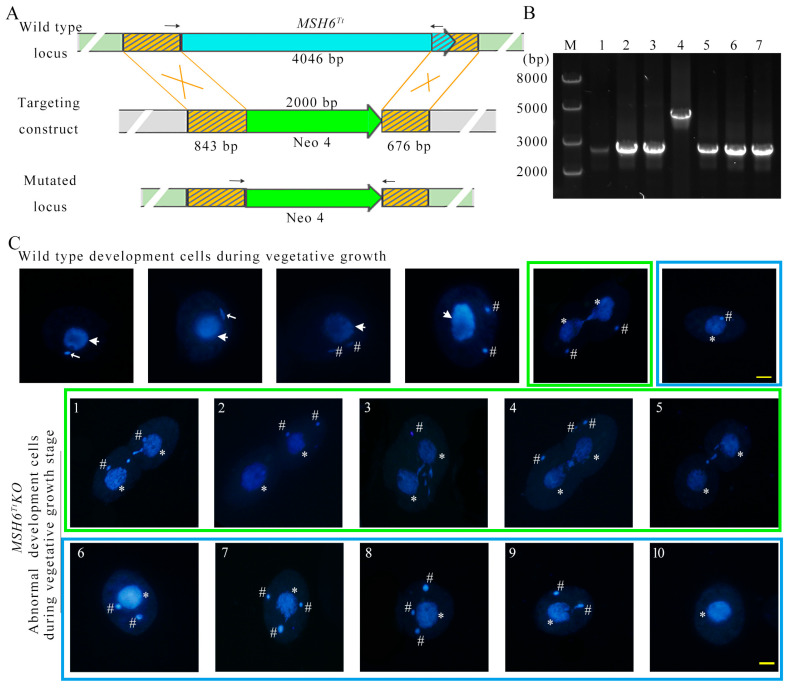
Knockout of *MSH6^Tt^* resulted in abnormal nuclear divisions in *Tetrahymena* during the vegetative growth stage. (**A**) Schematic representation for generating recombinant *MSH6^Tt^KO* mutants in *T. thermophila*. The blue arrow in the WT locus indicates the *MSH6^Tt^* gene; the yellow boxes with the black diagonal filling in the WT locus and targeting construct indicate the homologous arms. The *MSH6^Tt^* cassette is replaced by the Neo4 cassette at the mutated locus. The black arrows indicate the position of the PCR primer when identifying the mutant cell line (the length of the arrow is independent of the primer length). (**B**) The identification of *MSH6^Tt^KO*-B2086 and *MSH6^Tt^KO*-CU428 mutants. M indicates marker; 1–3 indicate *MSH6^Tt^KO*-CU428 mutants; 4 indicates WT cells; 5–7 indicate *MSH6^Tt^KO*-B2086 mutants. The WT and mutation sites were amplified by PCR; 2630 bp is for the mutation site, and 4327 bp is for the WT site. The black arrows in the figure (**A**) mark the positions of the primers. (**C**) *MSH6^Tt^* knockout affected nuclear division during vegetative growth. (**1**–**10**), The abnormal nuclear division of *MSH6^Tt^KO* mutants. The topmost part of the diagram shows normal nuclear development during the vegetative stage of WT in *T. thermophila*. The large white arrows indicate the MACs; the small white arrows indicate the MICs. # indicates the new MICs after mitosis; * indicates the new MACs after amitosis. DAPI staining of the nuclei is blue. The scale bar is 10 μm.

To explore the function of *MSH6^Tt^*, *MSH6^Tt^* was knocked out from the somatic genome of *T. thermophila* (Figure 4A). *MSH6^Tt^KO* mutants were confirmed by PCR (Figure 4B) and qRT-PCR (Figure S2, Table S3). After knockout of *MSH6^Tt^* in the somatic genome of *T. thermophila*, no expression of *MSH6^Tt^* was observed at vegetative growth and 2 h of conjugation. The new MACs developed from the zygotic nucleus during the late stage of conjugation. At this point, the *MSH6^Tt^* detected in the *MSH6^Tt^KO* mutants is the *MSH6^Tt^* expressed in the new MACs (Figure S2). Knockout of *MSH6^Tt^* did not affect the proliferation of *T. thermophila* (Figure S3(Ba)). However, the mutants showed cellular resistance to MMS (Figure S3(Ba)) and were sensitive to higher concentrations (4.5 mM) of cisplatin (DDP) (Figure S3(Bb,Bc)). After synchronization, the division indices of *MSH6^Tt^KO* and WT cells reached 62.16% and 56.39%, respectively (Figure S3(Aa)). However, 19.26% of *MSH6^Tt^KO* mutants showed abnormal nuclear divisions (Figure S3(Ab) and Figure 4(C1–C10)). Chromatin condensation after nuclear stretching was abnormal (Figure 4(C1,C3,C4,C5)). In the late M phase of MIC, the divided nucleus was not correctly distributed between the two daughter cells (Figure 4(C2–C4)), or the MIC was lost (Figure 4(C5)). Upon completion of nuclear division, the abnormal single cells exhibited one MAC with multiple MICs (Figure 4(C6–C9)) or without MICs (Figure 4(C10)).

### 2.5. MSH6^Tt^ Knockout Hinders Micronuclear Meiosis and Gametic Selection during the Sexual Development Stage 

**Figure 5:**
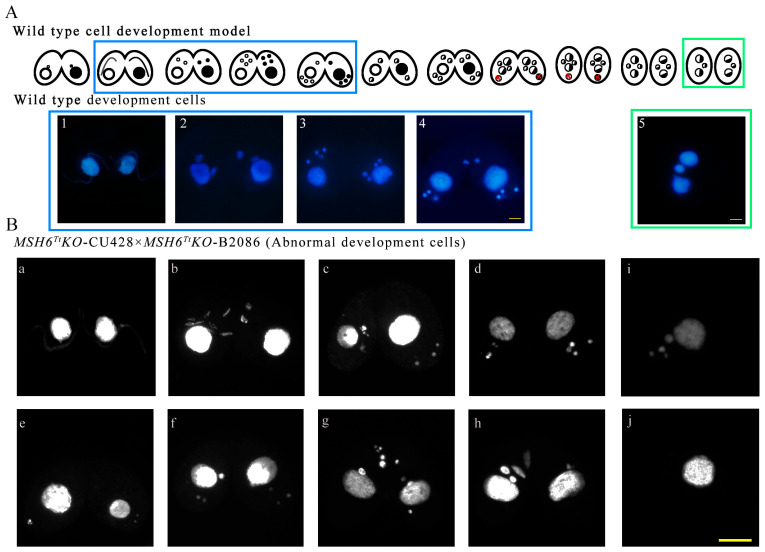
Abnormal nuclear divisions in the *MSH6^Tt^KO* mutant cell line during the conjugation stage. (**A**) The nuclear development during the conjugation stage in *T. thermophila*. (**1**), crescent elongating of MIC; (**2**), the first meiotic division (Meiosis Ι); (**3**), the second meiotic division (Meiosis II); (**4**), MIC “selection”; (**5**), Exconjugant with two MACs and one MIC. The topmost part of the diagram shows a diagram of the cell development model during the conjugation in *T. thermophila*. (**B**) Abnormal nuclear divisions in the *MSH6^Tt^KO* mutants during the conjugation stage. (**a**), crescent elongating of MIC; (**b**,**c**), MICs in *MSH6^Tt^KO* mutants were fragmented at the end of the first meiotic division; (**d**–**f**), meiotic products were stacked at the posterior of the cell; (**g**,**h**), meiotic products were stacked at the anterior of the cell; (**i**,**j**), abnormal single cells with multiple MICs (**i**) and without MICs (**j**). DAPI stains the nuclei. The scale bar is 10 μm.

The cell pairing and MIC elongation in the *MSH6^Tt^KO* mutants (Figure 5(Ba) and Figure S4) were consistent with those in WT (Figure 5(A1) and Figure S4). However, MICs in *MSH6^Tt^KO* mutants were fragmented at the end of the first meiotic division (Figure 5(Bb,Bc)) and were not consistent with the phenotype of WT (Figure 5(A2)). At 7 h of conjugation, 50.92% of *MSH6^Tt^KO* mutant cells were in meiosis, of which 60.00% showed abnormal nuclear developmental phenotypes (Figure S4). At the end of MIC selection, three MICs are located at the posterior of the cell, and one MIC remains at the anterior [35] (Figure 5(A4)). In *MSH6^Tt^KO* mutants, all meiotic products are located at the posterior of the cell (Figure 5(Bd–Bf)) or stacked at the anterior (Figure 5(Bg,Bh)) in *MSH6^Tt^KO* mutant cells. The abnormal single cells with multiple MICs and without MICs began to accumulate (Figure 5(Bi,Bj) and Figure S4). At 48 h of conjugation, only 11.50% of mutants developed into exconjugants with two MACs and one MIC, which decreased significantly compared with 57.97% for WT (Figure S4). The mutant phenotype was rescued when the *MSH6^Tt^KO* mutant mated with WT, and 33.82% of the cells completed sexual reproduction at 48 h of conjugation (Figure S4). The results indicated that Msh6^Tt^ affects micronuclear meiosis and gametogenesis and is needed for sexual development.

### 2.6. Msh6^Tt^ Interacts with Msh2^Tt^ and MMR-Independent Factors

**Figure 6:**
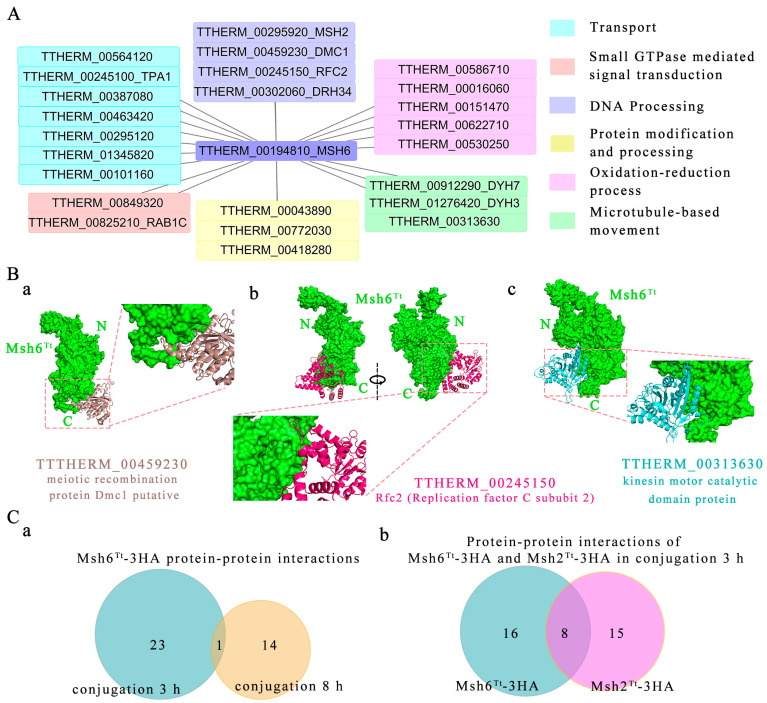
Msh6^Tt^-3HA interacts with both MMR-dependent factors and MMR-independent factors. (**A**) A protein interaction network map showing the proteins that interacted with Msh6^Tt^-3HA at 3 h of conjugation. The HA tag at the C-terminus of Msh6^Tt^-3HA was used to immunoprecipitate the Msh6^Tt^ interaction proteins in *T. thermophila*. WT cell lysates, where there was no HA tag, were also used to immunoprecipitate, with the protein obtained being used as a blank control in subsequent analyses and subtracted from the experimental group. The interacting proteins were identified by mass spectrometry analysis. MaxQuant was used to analyze mass spectrometry data. (**B**) Protein–protein docking results for (**a**) Msh6^Tt^ and Dmc1, (**b**) Msh6^Tt^ and Rfc2, (**c**) Msh6^Tt^ and TTHERM_00313630. (**C**) Msh6^Tt^ and Msh2^Tt^ have consistent and different interacting protein partners. (**a**) Msh2 ^Tt^ interacted with Msh6^Tt^-3HA at both 3 h and 8 h of conjugation. (**b**)The eight proteins interacted with both Msh6^Tt^-3HA and Msh2^Tt^-3HA at 3 h of conjugation. Msh2^Tt^-3HA protein interaction data were acquired from our previous data [34].

Coimmunoprecipitation (Co-IP) followed by mass spectrometry analysis was performed to identify interacters of Msh6^Tt^-HA at 3 h and 8 h of conjugation. To account for non-specific interactions or background noise, WT cell lysates without the HA tag were also subjected to the same Co-IP procedure using the HA antibody. The proteins obtained from this control group served as a blank control and were deducted from the experimental group. The presence of Msh6^Tt^-3HA after immunoprecipitation was verified using Western blot analysis (Figure S5). Raw data analyzed by MS were processed using MaxQuant, 24 (iBAQ WT/iBAQ Msh6^Tt^-3HA ≤ 0.05) (Figure 6A) and 15 (iBAQ WT/iBAQ Msh6^Tt^-3HA ≤ 0.1) (Table 1) interactors of Msh6^Tt^-3HA were identified at 3 h and 8 h of conjugation, respectively. The factors that interacted with Msh6^Tt^-3HA at 3 h of conjugation included Msh2^Tt^ (TTHERM_00295920) and MMR-independent proteins, such as Dmc1 (TTHERM_00459230), which participates in double-strand break repair during meiosis; Drh34 (TTHERM_00302060), which has ATP-dependent helicase activity and DNA binding activity; and replication factor C subunit Rfc2 (TTHERM_00245150), which is recognized as a carrier protein for Pcna [36]. The replication factor C subunit Rfc5 in yeast cells plays a direct role in sensing the state of DNA replication and signaling to checkpoint mechanisms [37]. the motor proteins Dyh3 (TTHERM_01276420), Dyh7 (TTHERM_00912290), and TTHERM_00313630 with microtubule dynamics activity; transporter-related ABC transporter family proteins (TTHERM_00564120); Tpa1 (TTHERM_00245100) and TTHERM_00387080, the P-type ATPase with catalytic activity and ATPase activity, also linked to the phosphorylation mechanism and transmembrane movement of ions; TTHERM_00463420, a protein with proton transmembrane transporter activity; TTHERM_00295120, a vacuolar protein sorting-associated protein 35 containing protein; TTHERM_01345820, a protein that is involved in intracellular protein translocation; TTHERM_00101160, a nucleoporin; endoplasmic reticulum membrane proteins TTHERM_00043890 and TTHERM_00772030 with transglycosylase activity; and TTHERM_00418280, a metalloendopeptidase, is involved in proteolysis. TTHERM_00586710, TTHERM_00016060, TTHERM_00151470, TTHERM_00622710, and TTHERM_00530250 are proteins with oxidoreductase activity that are involved in redox processes. TTHERM_00849320 and TTHERM_00825210_RAB1C are factors involved in signaling regulated by small GTPases (Figure 6A). Furthermore, protein–protein docking revealed that Dmc1 (Figure 6(Ba)), Rfc2 (Figure 6(Bb)), and the motor protein TTHERM_00313630 (Figure 6(Bc)) might have the ability to interact directly with Msh6^Tt^ (Figure 6B).

At 8 h after conjugation, the interacting factors with Msh6^Tt^ also included Msh2^Tt^ and MMR-independent factors, such as Rab11B (TTHERM_00941540); Gtu1 (TTHERM_00079520), which is involved in microtubule nucleation; TTHERM_01289180, TTHERM_00355660, and TTHERM_000079948, which are membrane proteins; TTHERM_01076960, a crooked neck-like protein that is involved in mRNA processing; RNA-binding protein Rrm53 (TTHERM_01099050); TTHERM_00085290, which is involved in metabolic processes with oxidoreductase activity; and TTHERM_00357080, a metal ion binding protein that exhibits superoxide dismutase activity (Table 1). The MMR-dependent factor Msh2^Tt^ was present in the interaction factors for both 3 h and 8 h of conjugation (Figure 6(Ca)).

Among the proteins that interacted with Msh2^Tt^-3HA at 3 h of conjugation [34], we also identified eight proteins that interacted with Msh6^Tt^-3HA (Figure 6(Cb), Table 2). All of these factors were involved in MMR-independent cellular processes, for example, TTHERM_00772030 and TTHERM_00043890, involving protein modification and processing; TTHERM_00622710 and TTHERM_00151470 are involved in oxidation-reduction processes; P-type ATPase TTHERM_00245100_*TPA1* is involved in cation transport; TTHERM_00387080, with catalytic activity and with ATPase activity, involving metabolic processes and coupling to transmembrane movement of ions; TTHERM_01276420, *DYH3* dynein heavy chain outer arm protein, involving microtubule-based movement; and TTHERM_00849320, which is involved in signaling regulated by small GTPase (Table 2).

### 2.7. Msh2^Tt^ Maintains the Stability of Msh6^Tt^

**Figure 7:**
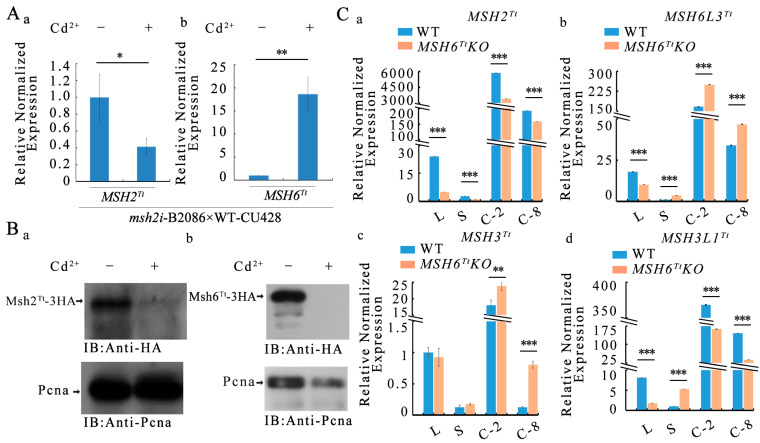
Expression of *MSH2^Tt^* maintains the stability of the Msh6^Tt^. (**A**) Knocking down *MSH2^Tt^* by adding Cd^2+^ in the *msh2i* mutants and WT mating pairs. The relative expression level of *MSH2^Tt^* (**a**) and *MSH6^Tt^* (**b**) in the mating pairs was detected by qPCR. The control was the gene expression level of *MSH2^Tt^* or *MSH6^Tt^* without Cd^2+^. Samples were collected at 4.5 h of conjugation. The *Y*-axis indicates the relative normalized expression of *MSH2^Tt^* (**a**) and *MSH6^Tt^* (**b**). Error bars represent the standard deviations for three replicates. * *p* < 0.05, ** *p* < 0.01. *p* values were calculated using Student’s *t*-test. (**B**) The expression level of Msh2^Tt^-3HA and Msh6^Tt^-3HA when the *msh2i* mutants mating with Msh2^Tt^-3HA or Msh6^Tt^-3HA mutants. (**a**), Western blot showed that the expression of Msh2^Tt^-3HA decreased after knocking down *MSH2^Tt^* by adding Cd^2+^ to the *msh2i* and Msh2^Tt^-3HA mating pairs. (**b**), The expression of Msh6^Tt^-3HA was not detected by Western blot after knocking down *MSH2^Tt^* by adding Cd^2+^ to the *msh2i* and Msh6^Tt^-3HA mating pairs. Samples were collected at 4.5 h of conjugation. The internal reference is Pcna. (**C**) The expression levels of *MSH2^Tt^* (**a**), *MSH6L3^Tt^* (**b**), *MSH3^Tt^* (**c**), and *MSH3L1^Tt^* (**d**) at different growth stages of *Tetrahymena* in WT cells and *MSH6^Tt^KO* mutants. L, vegetative growing cells; S, starvation cells; equal volumes of B2086 and CU428 cells were mixed, and samples were collected at 2 h and 8 h after mixing, referred to as C-2 and C-8. The *Y*-axis indicates the relative normalized expression of *MSH2^Tt^* (**a**), *MSH6L3^Tt^* (**b**), *MSH3^Tt^* (**c**), and *MSH3L1^Tt^* (**d**). Error bars represent the standard deviations for three replicates. ** *p* < 0.01, *** *p* < 0.001. *p* values were calculated using Student’s *t*-test.

The localization of Msh2 disappeared in human cancer cells with the knockout of *MSH6* [38]. In yeast cells, Msh2 and Msh6 form heterodimers in the cytoplasm and enter the nucleus as dimers. Msh6 deletion leads to a significant reduction in Msh2 in the nucleus. The Msh2-Msh6 dimer complex is also critical for the localization of Msh6 in the nucleus [39]. Msh6^Tt^ interacts with Msh2^Tt^ and may form heterodimers in *Tetrahymena* [34]. The *MSH2* knockdown *msh2i* mutants were created from our previous study [34]. In the *msh2i* mutants, *MSH2^Tt^* was knocked down by adding Cd^2+^. To further explore the relationship between Msh2^Tt^ and Msh6^Tt^ in *Tetrahymena*, we mated the *msh2i* mutants with WT cells. The transcription level of *MSH6^Tt^* was significantly increased (Figure 7(Ab), Table S4) in *MSH2^Tt^* knockdown mutants (Figure 7(Aa), Table S4). We also mated the *msh2i* mutants with the Msh2^Tt^-3HA mutants and the *msh2i* mutants with the Msh6^Tt^-3HA mutants. We found that Msh2^Tt^ deficiency (Figure 7(Ba)) affected the stability of Msh6^Tt^ (Figure 7(Bb)).

### 2.8. Expression of Redundant MSH6^Tt^ Family Genes

Msh3 redundantly performs the function of Msh6 in recognizing insertion/deletion mismatches in the Msh2-dependent mismatch repair mechanism in *Saccharomyces cerevisiae* [40]. Seven MutS homologous proteins are identified in *Tetrahymena*, with Msh6^Tt^, Msh6L3^Tt^, Msh3L1^Tt^, and Msh3^Tt^ clustered into a single unit in the phylogenetic tree [34]. The expression of *MSH2^Tt^* in *MSH6^Tt^KO* mutants was significantly decreased (Figure 7(Ca), Table S5). Knockout of *MSH6^Tt^* during the proliferation stage did not affect the expression of *MSH3^Tt^* (Figure 7(Cc), Table S5). The expression of *MSH6L3^Tt^* and *MSH3L1^Tt^* increased after *MSH6^Tt^* knockout during starvation (Figure 7(Cb,Cd), Table S5) but did not affect the expression of *MSH3^Tt^* (Figure 7(Cc), Table S5). The expression of *MSH6L3^Tt^* and *MSH3^Tt^* was significantly increased in the *MSH6^Tt^KO* mutant cell line at 2 h and 8 h of conjugation (Figure 7(Cb,Cc), Table S5). 

## 3. Discussion

The mismatch repair system repairs mismatches created during DNA replication and ensures the stability of chromatin [41]. The mutation of DNA mismatch repair genes has been observed in several human cancer cells. For example, the genes with mutations detected in hereditary nonpolyposis colorectal cancer cells are *MLH1* or *MSH2* [42]. Msh6 participates in a feedback loop, Msh6-CxcR4-Tgfb1, that promotes glioblastoma formation, proliferation, migration, and invasion [43]. Aberrant expression and transcriptional features of the *MSH6* have been observed in cancer cells [44,45,46]. Human Msh6 contains functional structural domains and an N-terminal disordered region. This disordered region contains motifs that interact with Pcna and H3K36me3 [10,47,48]. The N-terminal disordered region of *Tetrahymena* Msh6^Tt^ also has a conserved Pcna-interacting motif but does not possess a motif that interacts with H3K36me3 compared to human Msh6 [34]. The N-terminal disordered region exhibits differential functions in yeast and humans [30]. The disordered structural domains are associated with flexibility in protein function, thus ensuring adaptation to environmental stimuli [49]. Presumably, the function of the structural domain of the disordered N-terminal region of Msh6 converged toward complexity during eukaryotic evolution. Although *Tetrahymena* Msh6^Tt^ has a Pcna interaction motif at the N-terminus, we have not found that Msh6^Tt^-3HA interacts with Pcna in Co-IP-MS results. Msh6^Tt^-3HA interacted with Rfc2 (TTHERM_00245150) at 8 h of conjugation. Rfc loads Pcna onto DNA and subsequently leaves the Pcna-DNA complex by hydrolysis of ATP [36,50]. Pcna is involved in joining with Okazaki fragments, DNA repair, DNA methylation, and chromatin assembly, and is an essential protein in eukaryotic DNA replication and repair [51,52]. A large number of proteins in eukaryotes possess Pcna interaction motifs, and many of these proteins are involved in cellular signaling as hub proteins that interact with many other proteins [52]. Pcna functions as a scaffold protein in the cell. Loss of Msh6^Tt^ protein does not affect Pcna expression (Figure 7(Bb)). Presumably, Msh6^Tt^ is not an essential protein for Pcna function, and Pcna has instantaneous or weak interactions with Msh6^Tt^. A cancer-derived mutation located between the two nuclear localization signals of human Msh6 resulted in a significant reduction in the localization of hMsh6 in the nucleus, suggesting that an altered protein localization pattern may contribute to cancer [53]. In human cells, the four nuclear localization signals for hMsh6 are located between amino acids 246 and 313, and the localization of hMsh6 in the nucleus is remarkable [53]. In yeast cells, it has been observed that the nuclear localization of Msh6 is dependent on the presence of a functional nuclear localization sequence (NLS) in Msh2. When Msh2 lacks a functional NLS, the nuclear localization of Msh6 declines. This indicates that the formation of the Msh2-Msh6 dimer is crucial for the proper nuclear localization of Msh6. The dimerization of Msh2 and Msh6 likely facilitates the targeting and retention of Msh6 within the nucleus, highlighting the importance of this interaction in the cellular localization of Msh6 [39]. We found that Msh6^Tt^-3HA localized to the amitotic MAC and the mitotic MIC during vegetative growth and starvation in *T. thermophila*. It was shown that MMR is closely associated with cell replication in the S phase of nuclear division [54,55]. The distribution of fluorescent signals located in the perinuclear and intranuclear areas was uneven during the G2 and early M phases of MIC, and Msh6^Tt^-3HA moved from the perinuclear region of MIC into the MIC before the onset of mitosis, presumably correlating Msh6^Tt^ with replication during the S phase of MIC. We speculate that Msh6-3HA is stored in the nucleoplasm region or aggregated into heterochromatin prior to the onset of mitosis. The spindle-like localization of Msh6^Tt^-3HA during mitosis of the MICs suggested that it may be related to MIC stretching. In late mitosis, the middle part of the dumbbell-shaped structure contains no chromatin [35], but Msh6^Tt^-3HA is localized to the region, suggesting that Msh6^Tt^ is involved in chromatin segregation. Although knocking out *MSH6^Tt^* affected the nuclear division of MAC and MIC during vegetative growth, it did not significantly affect the vegetative proliferation of *Tetrahymena*. The MMR system also acts as a detector to detect DNA damage. The inactivity of the MMR subunit exhibits drug resistance [24]. MMS induces DNA fragmentation [56], which in turn affects the stability of the genetic material. The *MSH6^Tt^KO* mutants were tolerant to MMS but exhibited less response to DDP treatment. Msh6^Tt^ could sense DNA damage and differentially respond to different DNA damage reagents in *Tetrahymena*.

In *Tetrahymena*, the parental macronuclear genome does not replicate during conjugation [32]. Msh6^Tt^-3HA did not localize on the parental MAC in the mating cells. In contrast, Msh6^Tt^-3HA on the stretching meiotic MICs showed significantly stronger localization signals in the perinuclear region than in the central part of the nuclei. The fluorescence signal in the perinuclear region disappeared after detergent treatment. Msh6^Tt^ was not tightly bound to the perinuclear region. Msh6^Tt^-3HA formed a spindle-like structure in the first meiosis and the second meiosis of MIC. It was hypothesized that Msh6^Tt^ is associated with chromosome segregation during meiosis. *MSH6^Tt^* knockout led to abnormal micronuclear meiosis and gametic selection during the sexual development stage. DNA replication begins in the two developing new MACs immediately after mitosis of the zygotic nucleus has been completed. However, the replication does not take place in the two diploid MICs [57]. Msh6^Tt^-3HA localized in the two developing new MACs and disappeared from the parental MAC and new MICs. These results strongly suggest that Msh6^Tt^ is involved in DNA replication progress. Additionally, Msh6^Tt^ affects micronuclear meiosis and gametogenesis and is required for sexual development.

*MSH2^Tt^* knockdown affects the normal division of MIC and MAC during the vegetative growth of *Tetrahymena*, and affects proliferation capacity of *MSH2^Tt^KD* cells [34]. *MSH6^Tt^KO* mutants did not affect vegetative proliferation and had less effect on nuclear division than *MSH2^Tt^* knockdown mutants. This is possibly because the two distinct MutS heterodimeric complexes MutSα (Msh2-Msh6) and MutSβ (Msh2-Msh3) emerged during eukaryotic evolution [15]. Msh2 is needed for the formation of both complexes, and the function of Msh6 is made redundant by Msh3 in response to insertion/absence mismatches of single bases [15]. Msh3^Tt^, Msh3L1^Tt^, and Msh6L3^Tt^ are clustered with Msh6^Tt^ [34]. An interesting hypothesis is that *MSH6L3^Tt^*, *MSH3^Tt^*, and *MSH3L1^Tt^* in *Tetrahymena* might complement *MSH6^Tt^* deficiency at different stages of *Tetrahymena* development. *MSH3^Tt^* responded to the absence of *MSH6^Tt^* during vegetative proliferation; *MSH6L3^Tt^*, *MSH3L1^Tt^* and *MSH3^Tt^* functioned in *MSH6^Tt^KO* mutant strains during starvation; *MSH6L3^Tt^* and *MSH3^Tt^* were likely essential genes for cellular resistance to *MSH6^Tt^* knockout during conjugation. Further experiments are needed to explore these possibilities. These results partially explain why *MSH6^Tt^* knockout has less effect on nuclear division than Msh2^Tt^ knockdown in *Tetrahymena*.

The overall protein levels of Msh2 were significantly diminished in the absence of Msh6 in yeast [39]. In *Tetrahymena*, the decrease of *MSH2^Tt^* expression could be compensated by an increase in *MSH6^Tt^* expression at the transcript level. However, the absence of Msh2^Tt^ affects the stability of Msh6^Tt^. This effect on stability might be regulated through post-translational mechanisms, which ensure the proper functioning of the mismatch repair system. The stability of the Msh2^Tt^-Msh6^Tt^ heterodimer is higher compared to the monomeric member in *Tetrahymena*. This observation suggests a potential regulatory mechanism that could be conserved throughout eukaryotic evolution. The presence of stable heterodimers may play a role in maintaining the dynamic balance of monomeric mismatch repair proteins. 

Proteins interacting with Msh6^Tt^ at 3 h of conjugation include Msh2^Tt^ and MMR-independent factors. MMR-independent factors include Dmc1, which is involved in the repair of double-strand breaks; Rfc2, which is involved in the process of DNA replication and the deconjugating enzyme Drh34; Dyh3, Dyh7, and TTHERM_00313630, which are motor proteins with microtubule dynamics activity; and proteins with oxidoreductase activity. The “spindle-shaped” localization of Msh6^Tt^-3HA may be related to a functional interaction between Msh6^Tt^ and microtubule motor proteins. It has been reported that Dmc1 is a meiosis-specific protein and that deletion of Dmc1 in budding yeast leads to a significant reduction in meiotic DNA double-strand break repair, and that DNA double-strand breaks (DSBs) acquire longer single-stranded tails [58]. Dmc1 also plays an essential role in the process of homologous recombination in mammals [59]. In *T. thermophila*, Dmc1 is abundantly expressed in the meiotic nucleus [60]. Here, the possible interaction of Msh6^Tt^ and Dmc1 suggests that Msh6^Tt^ is involved in the meiotic process. The protein–protein docking results showed that Dmc1 might bind to the C-terminus of Msh6^Tt^. Furthermore, protein–protein docking results also suggested that Msh6^Tt^ and Rfc2 interact, which further supports our observations from immunofluorescent localization analysis that Msh6 is involved in DNA replication.

The proteins associated with Msh6^Tt^ at 8 h of conjugation included MMR-dependent and MMR-independent factors. Msh2^Tt^ was present in the interaction proteins of Msh6^Tt^-3HA at both 3 and 8 h of conjugation. The results indicated that Msh2^Tt^-Msh6^Tt^ functions as a stable heterodimeric complex during conjugation. The MMR-independent factors that interacted with Msh6^Tt^ at 8 h of conjugation are distinct from those at 3 h. It is hypothesized that Msh6^Tt^ functions differentially at distinct stages of conjugation. In addition, the eight proteins interacted with Msh2^Tt^ [34] as well as with Msh6^Tt^. These factors are involved in different MMR-independent cellular processes, including protein modification and processing, oxidation-reduction processes, cellular metabolism, microtubule motility, and small GTPase-regulated signalling. We hypothesize that Msh6^Tt^ and Msh2^Tt^ might participate concurrently in MMR-independent cellular processes. Some of these proteins may represent common contaminant proteins rather than bona fide interactions, further experiments are needed to explore these possibilities.

DNA mismatch repair serves as a conserved mechanism to ensure correct DNA replication. The MMR pathway mainly repairs post-replication base substitutions and insertion–deletion mismatches (IDLs) of bases [61]. Msh6^Tt^, as an essential protein in the mismatch repair mechanism, is involved in the amitosis of MAC, meiosis and mitosis of MIC, and DNA replication of the new MAC anlagen in *Tetrahymena*. There is tight interplay between Msh2^Tt^ and Msh6^Tt^. During eukaryotic evolution, Msh2 may have developed as a regulatory mechanism to maintain the stability of Msh6, ensuring a dynamic balance of monomeric mismatch repair proteins.

## 4. Materials and Methods

### 4.1. Cell Growth and Conjugation

*T. thermophila* strains B2086 (mating type II) and CU428 (mating type VII) were obtained from the *Tetrahymena* Stock Center (Cornell University, Ithaca, NY, USA). *Tetrahymena* cells were cultured in 1× SPP (Super Proteose Peptone) medium at 30 °C [62]. Cells were grown to the logarithmic phase (2.5–3 × 10^5^ cells/mL) and starved for 20~24 h in 10 mM Tris-HCl (pH 7.4) at 30 °C [63]. Conjugation was induced by mixing starved different mating type cells at equal densities.

### 4.2. Identification of Msh6^Tt^

The BLAST program of the human Msh6 and yeast Msh6 amino acid sequences was performed in the *T. thermophila* Protein MAC Genome Database (http://www.ciliate.org, accessed on 30 August 2022). The 3D modeling of Msh6^Tt^ was performed using Phyre2 [64].

### 4.3. Construction of Msh6^Tt^-3HA Mutants

The 696 bp sequence at the C-terminus of *MSH6^Tt^* and the 929 bp sequence downstream of *MSH6^Tt^* were amplified using *MSH6^Tt^*-3HA-5F/R and *MSH6^Tt^*-3HA-3F/R (Table S1), respectively. The two fragments were recombined with the pHA-Neo4 vector to obtain the pNeo4-*MSH6*-3HA recombinant plasmid. The detailed steps for plasmid construction and transformation were described previously [65]. The linearized plasmid fragments were transformed into different mating types of *Tetrahymena* cells using GJ-1000 (SCIENTZ, Ningbo, China) [65]. After screening for paromomycin resistance, the mutants were identified by PCR using the primer *MSH6*-3HA-Identify-F/R (Table S1).

### 4.4. Immunofluorescent Localization

Cells were fixed using 20% (*V*/*V*) paraformaldehyde and permeabilized using 10% (*V*/*V*) Triton X-100. The coverslips were blocked with blocking buffer (3% (*V*/*V*) Albumin Bovine V and 10% (*V*/*V*) Goat Serum dissolved in PBST (PBS plus 0.1% Tween-20)) for 1 h, followed by incubation with anti-HA primary antibody (1:500, Cell Signaling Technology, Danvers, MA, USA) for 2 h and FITC-conjugated anti-rabbit IgG secondary antibody (1:1000, Milliproe, Darmstadt, Germany) for 1 h. The cells were then incubated with 1 μg/mL DAPI for 10 min [60]. The cells were observed using the Delta Vision Elite microscope system (Applied Precision/GE Healthcare, Chicago, USA) or fluorescence microscope (BX51, OLYMPUS, Tokyo, Japan).

For cytological detection of chromatin-associated proteins, cells were treated with enhanced detergent spreading to determine whether the target protein is a chromatin-binding protein, and there will be no localization signals for the protein that is not chromatin binding [60]. Cell samples (5 mL) were taken, and 450 μL of 10% (*V*/*V*) Triton X-100 and 50 μL of 37% formaldehyde were added. After incubating for 30 min, 450 μL of 37% formaldehyde was added and incubated for 5 min at room temperature. After centrifugation at 1000× *g* for 2 min, the cells were collected and resuspended in 500 μL of the fixation solution (4% paraformaldehyde and 3.5% sucrose dissolved in ultrapure water), and 50 μL of cells was applied onto a polylysine-coated coverslip. The procedure for antibody incubation was the same as that for indirect immunofluorescence localization analysis.

### 4.5. Construction of MSH6^Tt^-Knockout Mutants

The 843 bp flanking sequence of *MSH6^Tt^* and the 676 bp sequence at the C-terminus of *MSH6^Tt^* were amplified by *MSH6KO*-5F/R and *MSH6KO*-3F/R (Table S1). The amplified fragments were sequentially recombined into the pNeo4 vector using the Hieff Clone^®^ Plus One Step Cloning Kit (Yeasen, Shanghai, China), and the pKO-Neo4-*MSH6* recombinant plasmid was obtained. The linearized plasmid fragments were transformed into *Tetrahymena* cells using GJ-1000 (SCIENTZ, Ningbo, China). The mutants were identified using the primer *MSH6KO*-Identify-F/R after the paromomycin gradient screen.

### 4.6. Synchronization of Cell Division

The cell cycle of *T. thermophila* is approximately 150 min at 30 °C. *Tetrahymena* undergoing temperature shock would arrest in the G2 phase of the MAC, which would complete the M phase within 15 min after the temperature returned to normal for 55–60 min [66]. Cells (0.1–0.5 × 10^5^ cells/mL) were incubated sequentially at 35 °C and 42 °C in a water bath for 30 min at each temperature, and after cycling 3–5 times, the cells were again placed at 35 °C in a water bath, and the cells began to divide in a synchronized manner after 50–60 min.

### 4.7. qRT-PCR Analysis

*Tetrahymena* cells (1 × 10^6^) were lysed with 1 mL of lysis solution (TRIeasy™ Total RNA Extraction Reagent, Yeasen, Shanghai, China) and then treated with chloroform, isopropanol, and ethanol to obtain total RNA. Single-stranded cDNA was synthesized according to the premix kit instructions (MonScript™ RTIII Super Mix with dsDNase (Two-Step) (Monad, Suzhou, China)), which is a two-step process involving degenomic and reverse transcription. The cDNA obtained above was used to prepare the reaction system according to the instructions of the Real-Time PCR Amplification Premix Kit (HieffTM qPCR SYBR Green Master Mix, Yeasen, Shanghai, China). The expression of *MSH2^Tt^*, *MSH6^Tt^*, *MSH6L3^Tt^*, *MSH3^Tt^*, and *MSH3L1^Tt^* in cells was analyzed via qRT-PCR using the primers *msh2i*-iden-F/R, *msh6i*-iden-F/R, q*MSH6L3*-F/R, q*MSH3*-F/R, and q*MSH3L1*-F/R (Table S1), respectively. The internal reference was 17S-F/R (Table S1).

### 4.8. Nuclear Development

A total of 3–3.5 × 10^5^ cells were collected at different developmental stages. Cells were fixed with 10 μL of 37% formaldehyde solution per 1 mL of sample and incubated with 0.1 μg/mL DAPI. The development nuclei were observed using fluorescence microscope (BX51, OLYMPUS, Tokyo, Japan).

### 4.9. Coimmunoprecipitation and Mass Spectrometry (Co-IP-MS)

*Tetrahymena* cells (3.5 × 10^7^) were collected at 3 h or 8 h of conjugation. The cells were lysed by sonication on ice in phosphate-buffered saline (PBS) buffer. Immunoprecipitation experiments were carried out using the Pierce™ HA-Tagged Magnetic IP/Co-IP kit (ThermoFisher, Waltham, MA, USA). The kit uses agarose beads coupled with high-affinity anti-HA antibodies to immunoprecipitate the HA-tagged protein and immunoprecipitate proteins that interact with the protein. The experiment process follows the instructions of the Co-IP kit. Wash solution: TBS (25 mM Tris, 0.15 M NaCl; pH 7.2), TBS plus 0.05% Tween-20 (TBS-T). The elution buffer is a 2× non-reducing sample buffer containing 0.12 M Tris-Hcl, 2% SDS, and 20% glycerol. Samples were digested using the protein endonuclease trypsin and then analyzed via LCMSMS (nanoLC-QE). Tandem mass spectra were obtained using a QE mass spectrometer based on the principle of higher-energy collisional dissociation. MaxQuant, quantitative proteomic analysis software, was used to analyze enormous mass spectrometry data [67]. Based on the iBAQ algorithm, the protein expression in the sample is approximated to be equal to the absolute concentration of the protein, and based on the protein expression intensity value of iBAQ intensity to screen the difference between the experimental group and the control, to identify the proteins that interacted with the HA-labeled protein [68]. Here, mass spectrometry data obtained after immunoprecipitation of WT cells without HA-tag were used as a control. Proteins with an iBAQ WT/iBAQ Msh6^Tt^-3HA ratio less than or equal to 0.05 (3 h of conjugation) or a ratio less than or equal to 0.1 (8 h of conjugation) were defined as proteins that have a specific intercalation relationship with Msh6^Tt^ in cells. Interaction network mapping of proteins was performed using Cytoscape [69].

### 4.10. Protein–Protein Docking

After synthesizing the amino acid sequences of the target proteins into a protein structure .pdb file using Phyre 2 [64], ClusPro was used to simulate protein interactions and obtain the interactions as .pdb files [70]. Finally, the interaction files obtained were observed using PyMOL, and the interactions of interest resulted in plots that were exported and optimized with PhotoShop 2020.

### 4.11. Western Blot Analysis

For immunoblotting detection of Msh2^Tt^-3HA or Msh6^Tt^-3HA in *Tetrahymena* paired cells, proteins were separated by 8% sodium dodecyl sulfate-polyacrylamide gel electrophoresis (SDS-PAGE). Subsequently, they were transferred to polyvinylidene fluoride (PVDF) membranes (Millipore, Shanghai, China). The blocking buffer, TBST (Tris-buffered saline buffer containing Tween-20) formulated with 5% skim milk powder, was used to block the PVDF membrane. The PVDF membrane was then incubated overnight at 4 °C with primary antibody (anti-HA, 1:500, Cell Signaling Technology, Danvers, MA, USA) diluted in blocking buffer. The membrane was then incubated with the HRP-conjugated secondary antibody (blocking buffer dilution, 1:1000, Sigma, Shanghai, China) for 1 h at room temperature. Immunoreactive bands were detected with EasyBlot ECL luminescent solution (Sangon Biotech, Shanghai, China) [71].

## Figures and Tables

**Table 1:** The proteins that interacted with Msh6^Tt^-3HA at 8 h of conjugation.

Gene Model Identifier	Gene Name	GO_Term
TTHERM_00372688	-	hypothetical protein
TTHERM_00268040	*NUP185*	
TTHERM_00112690	-	
TTHERM_00047280	-	
TTHERM_00941540	*RAB11B* (RAB GTPase 11B)	GTPase activity
TTHERM_00079520	*GTU1* (Gamma-TUbulin 1)	Microtubule nucleation
TTHERM_01289180	-	Integral component of membrane
TTHERM_00355660	-	
TTHERM_000079948	-	
TTHERM_01099050	*RRM53* (RNA recognition motif-containing protein 53)	RNA binding
TTHERM_01076960	-	mRNA processing
TTHERM_00085290	-	Oxidoreductase activity
TTHERM_00194810	*MSH6*	Mismatch repair
TTHERM_00295920	*MSH2*	
TTHERM_00418100	-	P28 protein
TTHERM_00357080	-	Superoxide dismutase

**Table 2:** The proteins that interacted with Msh6^Tt^-3HA and Msh2^Tt^-3HA at 3 h of conjugation.

Gene Model Identifier and Standard Name	GO_Term
TTHERM_00295920_*MSH2*	Mismatch repair
TTHERM_00194810_*MSH6*	
TTHERM_00772030	Protein modification and processing
TTHERM_00043890	
TTHERM_00622710	Oxidation-reduction process
TTHERM_00151470	
TTHERM_00387080	Cation transport/metabolic process
TTHERM_00245100_*TPA1*	
TTHERM_01276420_*DYH3*	Microtubule-based movement
TTHERM_00849320	Small GTPase mediated signal transduction

## Data Availability

All relevant data are within the paper and its additional files. The data used to support the findings of this study are available upon reasonable request.

## Supplementary Materials

The following supporting information can be downloaded at: https://www.mdpi.com/article/10.3390/ijms242417619/s1.
