# Supplementary material for: Mismatch Repair Protein Msh6Tt Is Necessary for Nuclear Division and Gametogenesis in Tetrahymena thermophila
Source: Int J Mol Sci. 2023 Dec 18;24(24):17619. doi: 10.3390/ijms242417619 (PMC10743813; doi:10.3390/ijms242417619)
Supplement: Supplementary file 1 [file ijms-24-17619-s001.zip › Supplementary Materials.pdf]

Figure S1. Western blotting analysis using whole cell lysates prepared from two different mating type *Tetrahymena* cells expressing $Msh6^{Tt}$-3HA. Lane 1 indicates untagged wild type mating cells; Lanes 2-4 indicate after equal volumes of $Msh6^{Tt}$-3HA-B2086 and $Msh6^{Tt}$-3HA-CU428 mutants were mixed, samples were collected at 5, 6, and 8 hours.

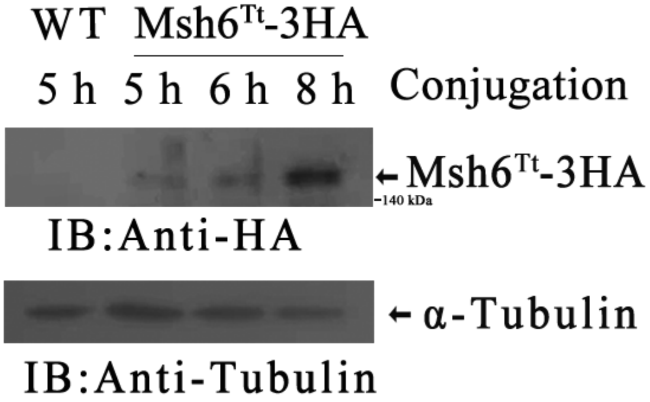

Figure S2. qRT-PCR was used to detect the relative expression levels of *MSH6*^{Tt} at different growth stages of *Tetrahymena* in wild-type cells and *MSH6*^{Tt}*KO* mutants. L, vegetative growing cells; equal volumes of B2086 and CU428 cells were mixed, and samples were collected at 2 h, 8 h and 16 h after mixing, referred to as C-2, C-8 and C-16. The Y-axis indicates the relative normalized expression of *MSH6*^{Tt}. The control was the gene expression level of *MSH6*^{Tt} in wild-type vegetative growing cells. Error bars represent the standard deviations for three replicates.

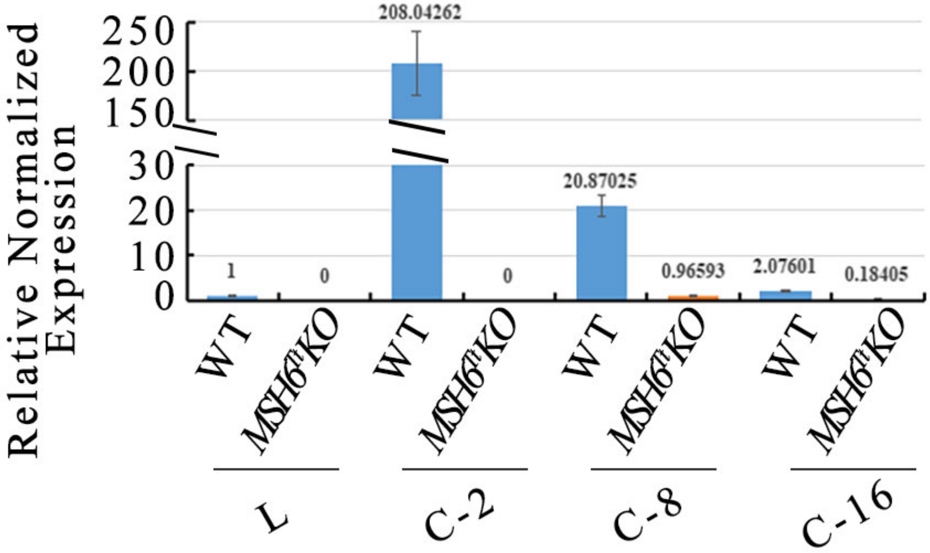

Figure S3. Knocking out *MSH6^{Tt}* resulted in abnormal nuclear division during vegetative proliferation in *Tetrahymena*. (A) a, Division index of *MSH6^{Tt}KO* cells and wild-type cells after five cycles of temperature gradient followed by 55-70 min; b, Nuclear development statistics of the nuclear division of the *MSH6^{Tt}KO* mutant cell line and wild-type control cells after synchronization during vegetative proliferation. The grey bars indicate the proportion of *MSH6^{Tt}KO* cells with abnormal nuclear divisions. The total number of cells counted was more than 460 for the mutant or wild-type cells. From the 55th minute behind five gradient temperature cycles, samples were taken every 5 minutes, totaling four times. At each time point, no less than 100 cells were counted. The topmost part of the diagram shows a diagram of the cell development model during vegetative growth of *T. thermophila*. (B) Vegetative growth curves of wild-type cells and *MSH6^{Tt}KO* mutant cells at 30°C for 48 h. The starting cell concentration was 0.125×10^5 cells/mL. a, Vegetative growth of *MSH6^{Tt}KO* cells versus wild-type cells within 48 h after and before treatment with 0.9 mM MMS. b, c, Vegetative growth of *MSH6^{Tt}KO* cells versus wild-type cells within 48 h after treatment with 0.9 mM DDP (b) and 4.5 mM DDP (c).

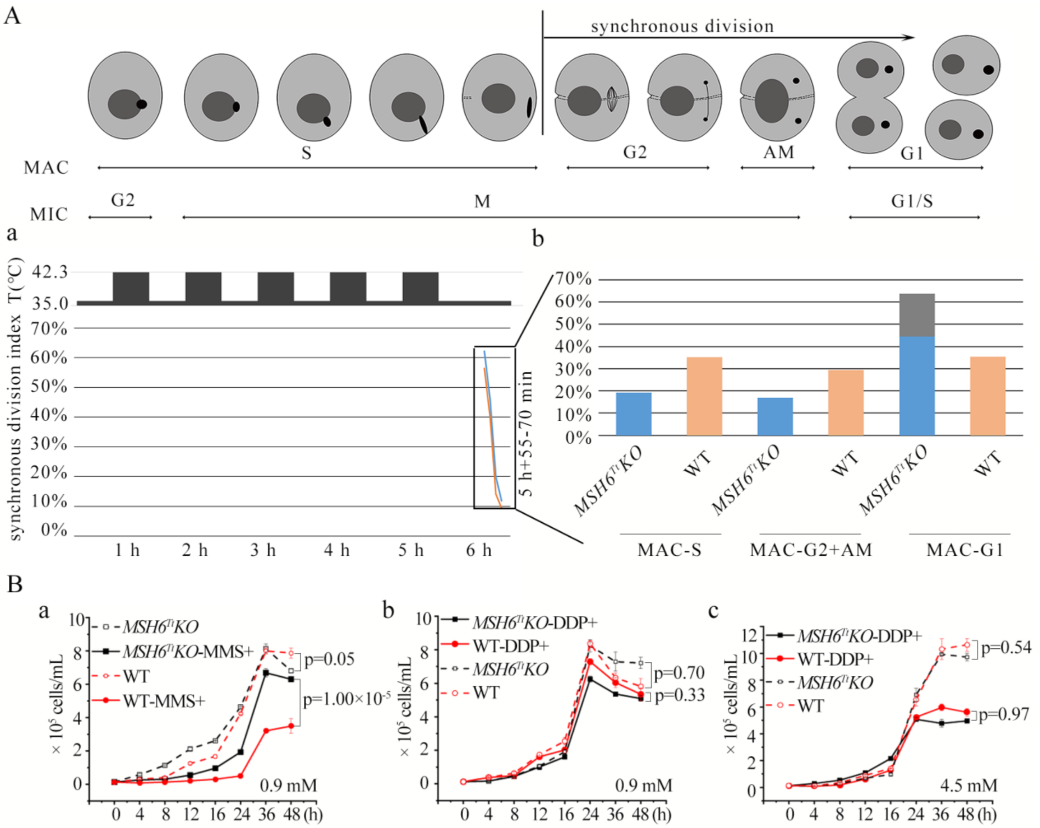

A
synchronous division
MAC
S
G2
AM
G1
MIC
G2
M
G1/S
a
b
T(°C)
42.3
35.0
synchronous division index
70%
60%
50%
40%
30%
20%
10%
0%
1 h
2 h
3 h
4 h
5 h
6 h
5 h+55-70 min
MSH6^Tt KO
WT
MAC-S
MAC-G2+AM
MAC-G1
B
a
MSH6^Tt KO
MSH6^Tt KO-MMS+
WT
WT-MMS+
p=0.05
p=1.00×10^-5
× 10^5 cells/mL
0.9 mM
0 4 8 12 16 24 36 48 (h)
b
MSH6^Tt KO-DDP+
WT-DDP+
MSH6^Tt KO
WT
p=0.70
p=0.33
0.9 mM
c
MSH6^Tt KO-DDP+
WT-DDP+
MSH6^Tt KO
WT
p=0.54
p=0.97
4.5 mM

Figure S4. Knocking out *MSH6*^{Tt} hinders the meiosis of MICs and the selection of gamete nuclei during the conjugation of *Tetrahymena*. The statistics of nuclear division of *MSH6*^{Tt}*KO* mutants and wild-type cells during conjugation. The number of cells counted was more than 200 for each time point and each cell line. The topmost part of the diagram shows a diagram of the cell development model and abnormal single cells during the conjugation of wild-type cells in *T. thermophila*.

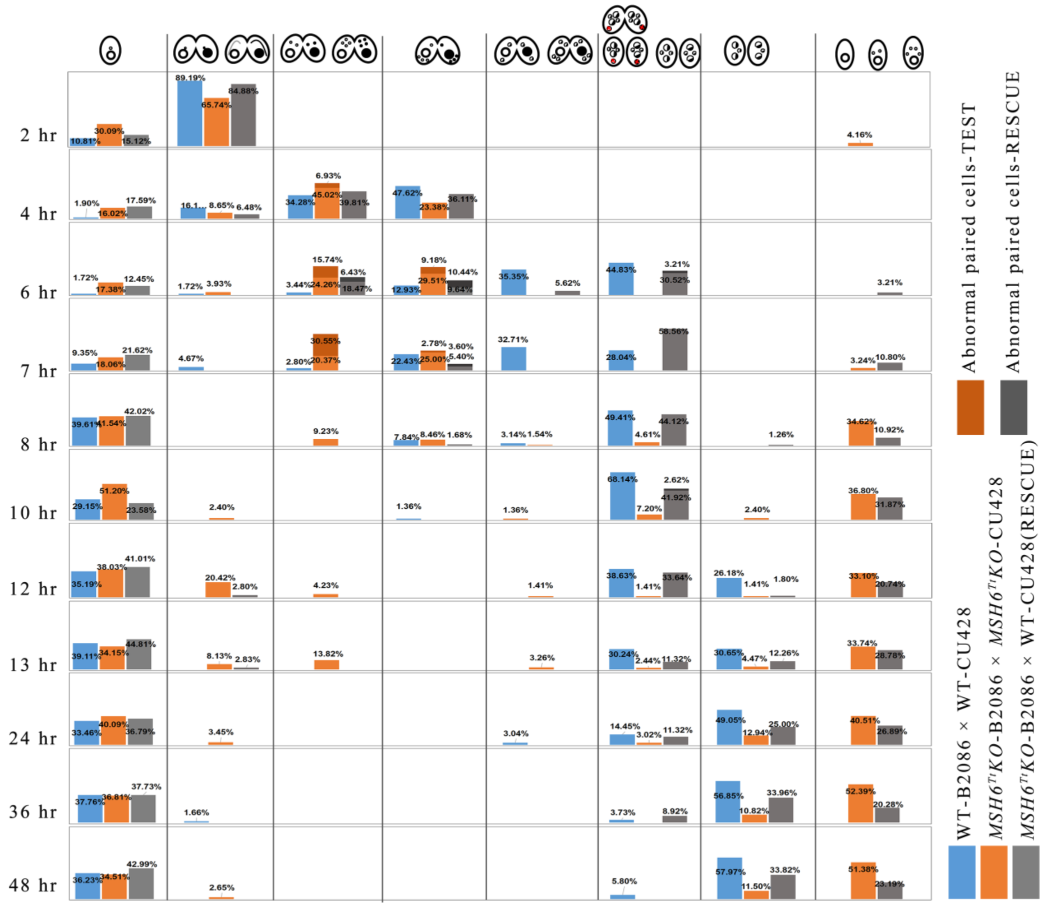

Figure S5. Western blots for the immunoprecipitation experiment. (A) Western blots for the IP experiment at 3 h of conjugation. (B) Western blots for the IP experiment at 8 h of conjugation. "IP: Anti-HA" indicates immunoprecipitation of HA-tagged proteins from whole cell lysates using anti-HA antibodies; IB: immunoprecipitation-blotting. The IB was probed with the indicated antibodies. Input: whole cell lysates before the IP experiment. WT: Untagged wild type cells; $Msh6^{Tt}$-3HA: Cells prepared from two different mating types *Tetrahymena* cells expressing $Msh6^{Tt}$-3HA.

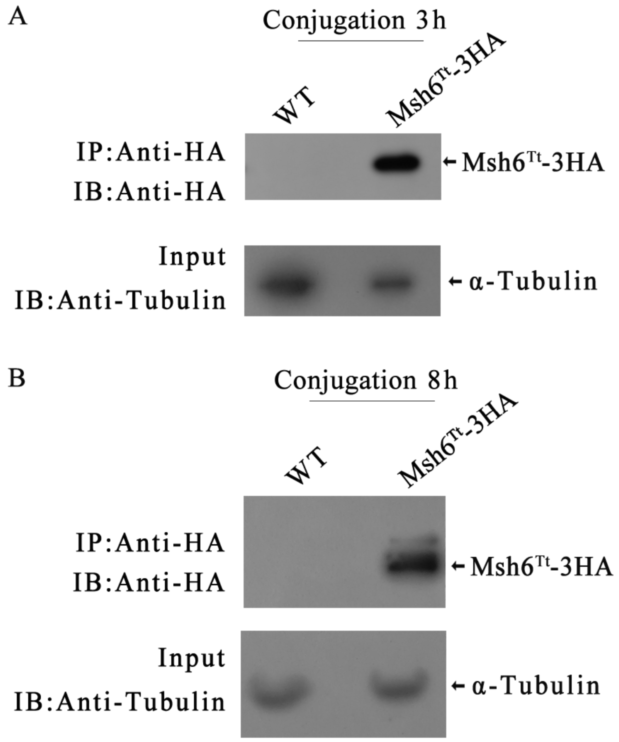

Table S1 Primer sequences used in the study.

Primer name	Primer sequences
MSH6-3HA-5F	GAGCTCTTAGCTTAAATTGGATGTTATGTACCTG
MSH6-3HA-5R	GCGGCCGCTAAAAGTTAAACTTTATTAAATTATTC AATAATT
MSH6-3HA-3F	CTCGAGTAAATAATAATAGAAATATATAATAGAC GCT
MSH6-3HA-3R	GGTACCTTATTGCTCTGTAATCAATTCGTTAACT
MSH6-3HA-Identify-F	ACATGGCATGTCATGTTGATGAGAAGAG
MSH6-3HA-Identify-R	ATTCTTATTCACTTTATGATTTTTATATTTG
MSH6KO-5F	CTAGAACTAGTGGATCCTCAATCATCGAATATCAT AAGCTTTAGTG
MSH6KO-5R	GGTACCCGGGGGATCCAAATTATTATTGTACTAAT CAATATATATG
MSH6KO-3F	CGATACCGTCGACCTCGACATGTCATGTTGATGAG AAGAGAAGT
MSH6KO-3R	CCGGGCCCCCCCTCGAGAACAATGAAAGGTAGTC AGTCATCTC
MSH6KO-Identify-F	TGCGCTCTCTGAATATCACTAAATATT
MSH6KO-Identify-R	ATCTCTATCATATTCTTAGATTGCTTTC
msh2i-iden-F	GACATTAATGTGAAAGAAAAGGATAAG
msh2i-iden-R	TAGACAGTTATAAATCACCTACAATAC
msh6i-iden-F	AAAGAATTGCGTGAACTCATAGCTG
msh6i-iden-R	CAAGAATATTAATTAAGGTATCCCATG
q*MSH6L3*-F	CATTAATGCTTGGACTAAGCTACTTG
q*MSH6L3*-R	CAATAAAAGACATCAAAGACCCTTCA
q*MSH3*-F	TCACGATCCTAAGTATCTCCTTGTT
q*MSH3*-R	CCTGGATCATAAACTATTTCTTAAGGT
q*MSH3L1*-F	GATAGAACATGTACAAAGCCAGGAG
q*MSH3L1*-R	CTTCGAAATAAACGCCTCTGCTCTT
17S-F	CCTGGGAAGGTACGGGTAAT
17S-R	AAGGTTCACCAGACCATTCG

Table S2. qPCR data of relative expression profiles of *MSH6*^{Tt} at different development stages.

Target	Sample	Expression	Expression SEM	Corrected Expression SEM	Mean Cq	Cq SEM
msh6i-iden-F/R	Li	6.43219	1.07369	1.07369	35.49	0.09309
	Lm	34.26583	4.00279	4.00279	32.76	0.16852
	Lh	48.40626	7.15288	7.15288	33.28	0.20583
	S0	44.147	6.22277	6.22277	33.91	0.20232
	S6	1	0.32648	0.32648	38.69	0.46421
	S12	7.45853	2.56155	2.56155	38.89	0.49538
	S24	0.6666	0.04631	0.04631	38.38	0.09122
	C0	7.88996	1.83547	1.83547	37.25	0.33154
	C2	4003.24516	249.8862	249.8862	28.65	0.08979
	C4	222.05318	21.03993	21.03993	31.04	0.10715
	C6	59.76434	10.46359	10.46359	32.3	0.24167
	C8	13.83002	1.97758	1.97758	34.32	0.20165
	C10	8.46318	2.57579	2.57579	35.11	0.29422
	C12	59.46812	19.5284	19.5284	32.54	0.47321
	C16	76.57617	10.79179	10.79179	33	0.20307
	C18	29.12	1.21301	1.21301	34.12	0.00165

Table S3. qPCR data of the relative expression levels of *MSH6*^{Tt} in wild-type cells and *MSH6*^{Tt}*KO* mutants at different growth stages.

**Target**	**Sample**	**Expression**	**Expression SEM**	**Corrected Expression SEM**	**Mean Cq**	**Cq SEM**
msh6i-iden-F/R	WT-L	1	0.20813	0.20813	32.44	0.23033
	WT-C-2	208.04262	31.87504	31.87504	26.04	0.21996
	WT-C-8	20.87025	2.40468	2.40468	29.66	0.11998
	WT-C-16	2.07601	0.26856	0.26856	33.01	0.17213
	MSH6^{Tt}*KO*-L	N/A	N/A	N/A	N/A	N/A
	MSH6^{Tt}*KO*-C-2	N/A	N/A	N/A	N/A	N/A
	MSH6^{Tt}*KO*-C-8	0.96593	0.17312	0.17312	33.85	0.23613
	MSH6^{Tt}*KO*-C-16	0.18405	0.03173	0.03173	36.58	0.24496

Table S4. qPCR data of the relative expression levels of *MSH2*^{Tt} and *MSH6*^{Tt} in the *msh2i* and WT mating pairs.

**Target**	**Sample**	**Expression**	**Expression SEM**	**Corrected Expression SEM**	**Mean Cq**	**Cq SEM**
msh2i-iden-F/R	Cd^{2+}-	1	0.27787	0.27787	31.24	0.382
	Cd^{2+}+	0.41289	0.09846	0.09846	31.94	0.31656
msh6i-iden-F/R	Cd^{2+}-	1	0.11089	0.11089	32.41	0.10398
	Cd^{2+}+	18.63234	3.62378	3.62378	24.78	0.11578

Table S5. qPCR data of the expression levels of *MSH2*^{Tt}, *MSH6L3*^{Tt}, *MSH3*^{Tt}, and *MSH3L1*^{Tt} at different growth stages of *Tetrahymena* in wild-type cells and *MSH6*^{Tt}*KO* mutant cell lines.

Target	Sample	Expression	Expression SEM	Corrected Expression SEM	Mean Cq	Cq SEM
msh2i-iden-F/R	WT-L	24.12896	0.00202	0.00202	34.89	0.10757
	MSH6^{Tt}*KO*-L	4.91839	0.00053	0.00053	36.34	0.12516
	WT-S	2.7261	0.00022	0.00022	38.01	0.17734
	MSH6^{Tt}*KO*-S	1	0.00008	0.00008	39.02	0.20869
	WT-C-2	5876.53927	0.39163	0.39163	28.41	0.17607
	MSH6^{Tt}*KO*-C-2	3434.43942	0.31028	0.31028	28.56	0.22881
	WT-C-8	192.42821	0.02891	0.02891	33.45	0.39628
	MSH6^{Tt}*KO*-C-8	158.03196	0.01794	0.01794	33.72	0.30076
q*MSH6L3*-F/R	WT-L	17.83801	0.06735	0.06735	32.76	0.17091
	MSH6^{Tt}*KO*-L	10.10324	0.01782	0.01782	33.13	0.0418
	WT-S	1	0.00663	0.00663	36.86	0.20713
	MSH6^{Tt}*KO*-S	3.56659	0.01532	0.01532	34.24	0.50909
	WT-C-2	166.90419	0.73118	0.73118	30.73	0.11681
	MSH6^{Tt}*KO*-C-2	249.9744	0.56374	0.56374	29.87	0.22676
	WT-C-8	33.89328	0.14057	0.14057	33.21	0.26051
	MSH6^{Tt}*KO*-C-8	46.73786	0.10597	0.10597	32.68	0.15283
q*MSH3*-F/R	WT-L	1	0.08282	0.08282	29.21	0.11223
	MSH6^{Tt}*KO*-L	0.92389	0.13912	0.13912	31.23	0.18214
	WT-S	0.12631	0.0328	0.0328	34.68	0.3239
	MSH6^{Tt}*KO*-S	0.17425	0.01758	0.01758	33.89	0.06055
	WT-C-2	18.00177	1.64022	1.64022	29.07	0.10291
	MSH6^{Tt}*KO*-C-2	23.94342	1.2691	1.2691	28.16	0.03826
	WT-C-8	0.12596	0.0129	0.0129	31.59	0.12865
	MSH6^{Tt}*KO*-C-8	0.80614	0.05561	0.05561	29.12	0.04687
q*MSH3L*1-F/R	WT-L	8.06583	0.01208	0.01208	35.45	0.15687
	MSH6^{Tt}*KO*-L	1.7261	0.00338	0.00338	36.83	0.20001
	WT-S	1	0.00217	0.00217	38.44	0.33835
	MSH6^{Tt}*KO*-S	5.2964	0.00857	0.00857	35.6	0.27023
	WT-C-2	368.65086	0.33641	0.33641	31.39	0.15078
	MSH6^{Tt}*KO*-C-2	162.57124	0.10187	0.10187	31.94	0.07318
	WT-C-8	145.78738	0.1633	0.1633	32.84	0.17836
	MSH6^{Tt}*KO*-C-8	37.48674	0.25864	0.25864	34.77	1.15305
